# AMP-activated protein kinase — a journey from 1 to 100 downstream targets

**DOI:** 10.1042/BCJ20220255

**Published:** 2022-11-16

**Authors:** D. Grahame Hardie

**Affiliations:** Division of Cell Signalling & Immunology, School of Life Sciences, University of Dundee, Dow Street, Dundee DD1 5EH, Scotland, U.K.

**Keywords:** history, intracellular signaling, phosphorylation/dephosphorylation, protein-serine–threonine kinases

## Abstract

A casual decision made one evening in 1976, in a bar near the Biochemistry Department at the University of Dundee, led me to start my personal research journey by following up a paper that suggested that acetyl-CoA carboxylase (ACC) (believed to be a key regulatory enzyme of fatty acid synthesis) was inactivated by phosphorylation by what appeared to be a novel, cyclic AMP-independent protein kinase. This led me to define and name the AMP-activated protein kinase (AMPK) signalling pathway, on which I am still working 46 years later. ACC was the first known downstream target for AMPK, but at least 100 others have now been identified. This article contains some personal reminiscences of that research journey, focussing on: (i) the early days when we were defining the kinase and developing the key tools required to study it; (ii) the late 1990s and early 2000s, an exciting time when we and others were identifying the upstream kinases; (iii) recent times when we have been studying the complex role of AMPK in cancer. The article is published in conjunction with the Sir Philip Randle Lecture of the Biochemical Society, which I gave in September 2022 at the European Workshop on AMPK and AMPK-related kinases in Clydebank, Scotland. During the early years of my research career, Sir Philip acted as a role model, due to his pioneering work on insulin signalling and the regulation of pyruvate dehydrogenase.

It was an honour and a privilege to be asked to present one of the Sir Philip Randle lectures of 2023. I was lucky enough to meet Sir Philip on several occasions and looked up to him in more ways than one. Firstly, in the metaphorical sense, since he was a UK pioneer in the field of cell signalling that I aspired to enter, who had made important contributions to insulin signalling and secretion, and also to regulation of metabolism, particularly of pyruvate dehydrogenase. Secondly, I looked up to him in the literal sense: I had always thought of myself as being tall at 6′ 6″ or 2 m, but in that respect I had to defer to Sir Philip! There is also an indirect connection between Sir Philip and the growth of research in cell signalling at my own institution, the University of Dundee. In 1964 Philip had moved from Cambridge to Bristol University to become their first Professor of Biochemistry, and very rapidly established it as a centre of excellence. One of several people he persuaded to move with him from Cambridge to Bristol was Peter Garland, a medical graduate who had switched to science and who, during his PhD, had worked on what became known as the Randle cycle. In 1970, Peter was appointed as the first Professor of Biochemistry at Dundee and immediately did a ‘Randle' by persuading several people, including David Boxer and Bruce Haddock, to move from Bristol to Dundee, which some biochemists might have considered at that time to be the ‘back of beyond'! However, perhaps the most important appointment he made was that of Philip Cohen, who had just completed a postdoc with Ed Fischer, one of the fathers of the protein phosphorylation field. Although Philip Cohen had only published a few papers at that time, Peter Garland clearly recognized his potential as a rising star who was to firmly establish the reputation of Dundee in the cell signalling field. Thus, it is easy to see the legacy of Philip Randle, via his student Peter Garland, in putting Life Sciences at my own University on the map.

Since it is not every day that one gives a Sir Philip Randle lecture, and since I am nearing retirement and thus the end of my own 50 year journey in research, I hope I will be forgiven for indulging in this review in some reminiscences of that journey. I will deal with three periods of my studies on AMP-activated protein kinase (AMPK), each separated by around 20 years, starting with my earliest steps into the story.

## Early days — defining the AMP-activated protein kinase

In October 1975, I moved to Dundee from a postdoc position at what was then Portsmouth Polytechnic, where I had been studying protein kinases that phosphorylated histone H1 in the slime mould *Physarum polycephalum*. This organism can grow as an acellular plasmodial form that is a *coenocyte*, in which a single common cytoplasm several centimetres across can contain hundreds of millions of nuclei, which enter mitosis with perfect synchrony. The activity of the H1 kinases varied dramatically during the plasmodial cell cycle, peaking just before mitosis and then disappearing [1,2] and with hindsight it is obvious that they were CDK/cyclin complexes, although we didn't know that at the time (in a landmark review in 1990, Paul Nurse was kind enough to point out that these studies in *P. polycephalum* had preceded other research on kinases controlling the cell cycle ‘by nearly 15 years' [3]). In Dundee, I had started a second postdoc that also involved protein phosphorylation [4], but it soon became clear to me that it was Philip Cohen's laboratory that was really going places. The following year an opportunity arose when Philip obtained a Fellowship from the Wellcome Trust enabling him to focus on research, with the grant paying for a temporary replacement on the teaching staff, to which I was appointed. Since this was only for 2 years initially, Peter Garland said that he could not set me up in my own laboratory, and that I should occupy some of Philip Cohen's space instead. I was happy with that arrangement since I knew I would benefit not only from Philip's advice, but also from gaining access to his specialist equipment and library of reagents, such as purified protein kinases and phosphatases. Moreover, the position did give me the opportunity to start my own research project at the age of only 26.

The first thing was to decide was what I should work on. One evening towards the end of 1976 Philip Cohen and I went down to a bar near the Biochemistry Department to discuss this. It was obvious that I would work on something to do with protein phosphorylation, but on what exactly? It is hard to believe now, but at that time the only process where the role of protein phosphorylation was reasonably well understood was glycogen metabolism. However, Philip already worked on that, so I wanted to work on something different. I'd like to be able to say that I spent days or weeks agonizing about what this should be, but it wasn't like that at all. Philip had brought along to the bar 3 or 4 papers describing preliminary evidence that processes other than glycogen metabolism might be regulated by protein phosphorylation. By the end of our second beer I had made a snap decision to follow up a paper from Ki-han Kim at the University of Indiana [5] concerning acetyl-CoA carboxylase (ACC), which catalyzed a rate-limiting step in fatty acid synthesis. Kim had shown that a crude preparation of ACC from rat liver became activated over time when incubated with Mg^2+^, an effect that was blocked by fluoride ions; conversely, ACC was inactivated in a time-dependent manner when incubated with Mg.ATP^2−^ in the presence of another crude protein fraction, somewhat provocatively called fraction K. Since it was already known that many protein phosphatases were metal ion-dependent and inhibited by fluoride, while protein kinases required Mg.ATP^2−^ as co-substrate, Kim surmised that ACC was being inactivated by phosphorylation by a protein kinase present in fraction K. While the evidence for that was reasonably convincing, the kinase in fraction K was completely uncharacterized, and I resolved to pursue it.

One of Philip Cohen's mantras at that time was ‘don't waste clean thinking on dirty enzymes', a saying originally attributed to the eminent biochemist Efraim Racker. Philip's initial advice was therefore to start by purifying ACC to homogeneity, and to achieve this by purifying it from the richest source I could find. A literature search revealed that, because milk has a high fat content, lactating mammary gland was an extremely rich source of enzymes of fatty acid synthesis, so I started using mammary gland from lactating rabbits, although we later switched to rats. This turned out to be an excellent choice, because within a few weeks I had worked out a simple procedure to purify ACC for use as a substrate, as well as a kinase assay that measured incorporation of radioactivity from [γ-^32^P]ATP into the purified protein [6].

Our ACC preparation, even though apparently homogeneous by SDS–PAGE, contained traces of at least two protein kinase activities that phosphorylated ACC, one of which appeared to be the well-known cyclic AMP-dependent protein kinase (PKA) and another that we called ACC kinase-2 or ACK2 [6]. Armed with these preliminary results, with Philip's help I was successful in obtaining a project grant from the MRC (Medical Research Council), giving me financial independence and allowing me to appoint my first postdoc, Paul Guy. Meanwhile, Philip had obtained renewed MRC funding for his own research that covered his salary. This meant that my own position was now secured for at least 5 years, and Peter Garland finally agreed to set me up in my own laboratory, in a former teaching laboratory with plenty of room for expansion.

Phosphorylation of ACC by PKA proved to be a rather lengthy false trail or ‘red herring'. Treatment of adipocytes [7] or hepatocytes [8] from rats with the cyclic AMP-elevating hormones adrenaline or glucagon, respectively, led to phosphorylation of ACC within the same tryptic or chymotryptic peptides that contained the major site phosphorylated on purified ACC by PKA, suggesting that the latter kinase was responsible. However, it turned out later that the serine residues phosphorylated within those peptides in intact cells treated with PKA activators, and in cell-free assays by PKA, were different (see below) [9]. These studies, which were before the development of phosphospecific antibodies, required the labelling of cells with heroic amounts of ^32^P-phosphate, and were performed by myself with Roger Brownsey (a PhD student in Bristol with Dick Denton, the latter another protégé of Philip Randle) [7], by my first PhD student, Ross Holland, with the help of a sabbatical visitor to my laboratory from the US, Lee Witters [8], and later on by my postdoc Alastair Sim [9].

**Figure 1.**
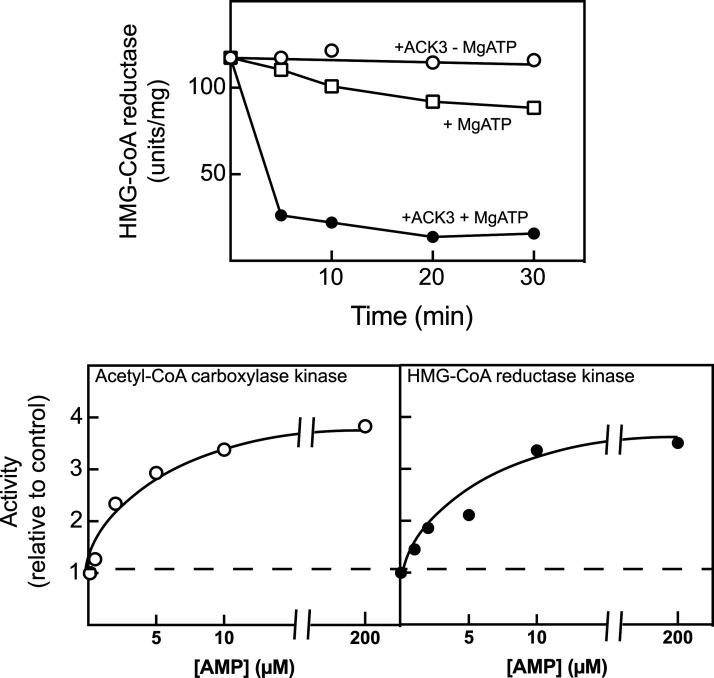
Some of the original evidence that ACC kinase and HMGCR kinase were the same entity. Top: An acetyl-CoA carboxylase kinase (ACK3, now known as AMPK) purified by 700- to 1000-fold from rat liver inactivated HMG-CoA reductase from Chinese hamster UT-1 cells in a time- and MgATP-dependent manner (the small degree of inactivation in the absence of ACK3 was most likely due to a small amount of endogenous AMPK in the HMGCR preparation, which was a crude membrane extract from UT-1 cells). Bottom: the acetyl-CoA carboxylase kinase (left) and HMG-CoA reductase kinase (right) activities of ACK3 were allosterically activated by AMP in a similar manner. Graphs redrawn from [20].

While in Philip Cohen's laboratory I had purified ACC from rabbit mammary gland in the presence of the protein phosphatase inhibitor fluoride, resulting in a preparation that had low specific activity. However, when fluoride was removed and the preparation was incubated with a protein phosphatase, there was a large activation [10]. Paul Guy and I found [11] that while incubation with MgATP^2−^ and either PKA or ACK2 could partially reverse this activation, neither kinase could fully reverse it, which implied the existence of a third kinase with a much larger effect on ACC activity; we began to call this ACC kinase-3 or ACK3. In 1984 a new PhD student, Dave Carling, started in the laboratory, and I set him the task of purifying ACK3. In the summer of 1986 I spent several weeks working in the U.S.A. on the regulation of tyrosine hydroxylase by phosphorylation [12]. When I returned to Dundee I was not expecting that Dave would have made much progress while I was away, but in fact he had an extremely exciting finding! We had decided at the outset that we would purify ACK3 in the presence of fluoride, not because we thought that the kinase would be regulated by phosphorylation, but to inhibit protein phosphatases that might interfere with our kinase assays — this turned out to be crucial. Dave had wanted to try hydroxyapatite chromatography as a potential purification step for ACK3, but this had to be performed in the presence of Ca^2+^ so he had to dialyze away the fluoride ions, which would otherwise cause precipitation of calcium fluoride. However, to his great surprise, when he did this he lost all ACK3 activity, although if he dialyzed in the presence of fluoride it was quite stable. We immediately realized that ACK3 must itself be inactivated by dephosphorylation, and it made us realize that it might be related to another poorly characterized kinase that phosphorylated and inactivated HMG-CoA reductase (HMGCR, which catalyzed what was thought to be a rate-limiting step in cholesterol synthesis). This putative kinase activity had been first described in a crude rat liver extract by David Gibson's group in Indianapolis in 1973 [13]. In 1978 Tom Ingebritsen, a PhD student from that group, had provided evidence that the HMGCR kinase was itself inactivated by dephosphorylation (just like our ACK3), and could be reactivated by treatment with MgATP^2-^ and another fraction that was presumed to contain an upstream kinase [14]. I knew all about those results because, after finishing his PhD, Tom had moved to Dundee to become a postdoc in Philip Cohen's laboratory, where we had been neighbours. It soon became obvious that our ACK3 was probably identical not only with the ACC kinase of Kim [5] but also with the HMGCR kinase of Gibson [13]. For example, Kim had reported that his ACC kinase was allosterically activated by AMP [15], and this was later also reported for HMGCR kinase by Hegardt's group in Spain [16]. To test whether our purified ACK3 also acted on HMGCR, we needed a source of the latter enzyme and an assay. Following once again Philip Cohen's advice to use the most abundant source to purify an enzyme, I wrote a letter (this was before the era of e-mails) to Joseph Goldstein, who with Michael Brown had just won the Nobel Prize in Physiology or Medicine for their work on cholesterol metabolism. One thing they had done was to generate UT-1 cells, in which the *HMGCR* gene had been amplified, and expression of HMGCR protein increased 500-fold, due to several rounds of selection for resistance to compactin, an early example of the class of HMGCR inhibitors now known as statins [17]. Goldstein and Brown had in fact also published a couple of papers on HMGCR kinase [18,19], and I had a slight concern that they might simply do the experiments I had proposed in my letter by themselves. However, I need not have worried because Goldstein quickly replied and sent us the cells, for which I will be eternally grateful. HMGCR is embedded in the ER membrane and can be tricky to work with, but it was so abundant in UT-1 cells that I could set up a simple spectrophotometric assay using a crude membrane fraction, monitoring NADPH oxidation in the presence of HMG-CoA; this was much simpler than the more commonly used radiochemical assays. We could show that Dave's ACK3, after purification by 700- to 1000-fold from rat liver, would inactivate both ACC and HMGCR, and that both activities were allosterically activated by AMP and sensitive to protein phosphatase treatment in very similar ways (Figure 1).

I knew that these were important findings and sent a manuscript to Nature, who rejected it without sending it for review! As I was terrified that we would be ‘scooped', I immediately sent it instead to FEBS Letters, which had a reputation for rapid publication. Many years later I was gratified to see that, in the ‘Timeline of Physiology' created for the 125th Anniversary of the American Physiology Society in 2012, this paper [20] was cited as one of the most important discoveries of the year 1987.

**Figure 2.**
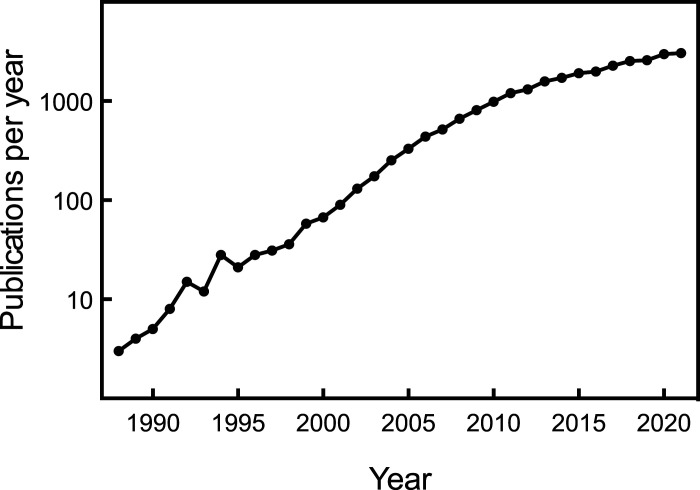
Number of papers that mentioned AMPK published per year between 1988 and 2021. For each year, PubMed was searched for the presence of the terms ‘AMPK' or ‘AMP-activated' in any field. Note that the y axis is logarithmic.

Thus, ACK3 now had 2 targets rather than 1, and before long we had identified two more (glycogen synthase [21] and hormone-sensitive lipase [22]). As there seemed no reason for it to stop there (as the title of this article suggests, the number of targets for AMPK now stands at more than 100), I thought that the names ACC kinase or HMGCR kinase were no longer appropriate. We therefore rather presumptuously renamed it AMPK after its allosteric activator, AMP — fortunately for us, this name has stuck. From our first use of the term in 1988 [9,23], the number of papers that mention AMPK per year has grown inexorably, passing 10 in 1992, 100 in 2002 and 1000 in 2011 (Figure 2).

Other important developments were taking place in the laboratory at this time. My postdoc Mike Munday identified, by amino acid sequencing of peptides, two sites on ACC phosphorylated by AMPK [23]. When Ki-han Kim published the complete sequence of rat ACC predicted from the cDNA sequence later the same year [24], these could be identified as Ser79 and Ser1200, while a third site we later showed to be Ser1215 [25]. Interestingly, PKA phosphorylated a different serine residue, Ser77, within the same tryptic or chymotryptic peptide as Ser79 [9], which is why we had previously mistakenly thought that PKA was phosphorylating ACC in intact cells [7,8]. It soon became clear that phosphorylation of Ser79, rather than Ser1200 or Ser1215, accounted for most of the inactivation of ACC caused by AMPK [23,25]. This yielded three major benefits that we were able to capitalize upon:

(1) My PhD student Steve Davies developed the *SAMS* peptide, a synthetic peptide based on the sequence around Ser79 (with the PKA site at Ser77 changed to alanine), for use as a substrate in AMPK assays [26]. One small batch of synthesis of this peptide turned out to be sufficient for thousands of assays, obviating the need for the time-consuming, twice-monthly purification of ACC that we had previously used as substrate for our kinase assays. This assay also made it easier for other laboratories to study AMPK, and variants of it were later used by pharmaceutical companies to successfully search for novel AMPK activators in high-throughput screens [27–29].
(2) My PhD students John Weekes and Susan Dale used variants of the *SAMS* peptide [30] and a related peptide [31], based on the sequences around sites on the four known targets [21,22,25,32], to establish the motif via which AMPK recognizes its target sites — this recognition motif has been very helpful in identifying the >100 sites for AMPK that we know about today (a list of which has been compiled in a recent review [33]).
(3) We later generated phosphospecific antibodies against this site [34] — monitoring of Ser79 phosphorylation on ACC using phosphospecific antibodies remains an almost universally used marker for AMPK activation in intact cells and *in vivo*.

## The 1990s and early 2000s — identifying upstream kinases

In 1994 our group [35] and Bruce Kemp's group in Melbourne [36] reported the complete purification of AMPK, revealing that it contained three subunits, now termed α, β and γ, in a 1:1:1 complex. We had previously identified the largest (α) subunit as the catalytic subunit using an affinity labelling approach [37]. Meanwhile, Dave Carling had moved to the laboratory of James Scott in London where, working in collaboration with Raj Beri at Zeneca Pharmaceuticals (now AstraZeneca) he managed to clone cDNA encoding an α subunit of AMPK [38]. Based on DNA [38] and protein [36] sequences respectively, Dave and Bruce Kemp recognized that the α subunit was the mammalian orthologue of Snf1, the catalytic subunit of a kinase shown a few years earlier by Marian Carlson to be required for responses to glucose starvation in the yeast *Saccharomyces cerevisiae* [39]. Bruce also showed [40] that the other two subunits (now β and γ) were related to Sip1/Sip2/Gal83 and Snf4, respectively, which interacted with Snf1 [41–43] to form what we now call the SNF1 complex. Subsequently, Bruce's group cloned a second mammalian α subunit that they called, a little perversely, α1, with the isoform cloned earlier by Dave Carling becoming α2 (Dave quipped that because Australians were in the southern hemisphere and therefore upside down relative to the UK, perhaps they counted backwards?). Cloning by Dave's and Bruce's groups of cDNAs encoding two isoforms of the β subunit (β1, β2) and three of the γ subunit (γ1, γ2, γ3) soon followed [44–47]. My group also provided the first evidence, using a reactive AMP analogue, that the γ subunit contained the allosteric binding site for AMP [46].

Why should AMPK be allosterically activated by AMP? We had shown that the effect of AMP was antagonized by high concentrations of ATP [37], so the kinase effectively monitored the cellular AMP:ATP ratio. The major source of cellular AMP is thought to be the reaction catalyzed by adenylate kinase (2ADP ↔ ATP + AMP), which has an equilibrium constant close to 1. If this reaction is operating close to equilibrium (which appears to be the case in most cells), it is easy to show that the AMP:ATP ratio will vary as the square of the ADP:ATP ratio [48], making the former a much more sensitive indicator of falling energy status than the latter. Incidentally, I was very pleased with myself when I worked out this line of argument, only to find that Sir Hans Krebs had come to the same conclusions almost 40 years earlier [49], when he stated; ‘this means that the absolute concentration of AMP is a much more sensitive controlling agent than the absolute concentration of ADP, or of ATP, or of the ADP:ATP ratio'. Personally, I think it is revealing that three other enzymes that also directly monitor cellular energy status, i.e. glycogen phosphorylase, fructose-1,6-bisphosphatase and phosphofructokinase all (like AMPK) appear to respond primarily to AMP and ATP, rather than to ADP and ATP [50].

**Figure 3.**
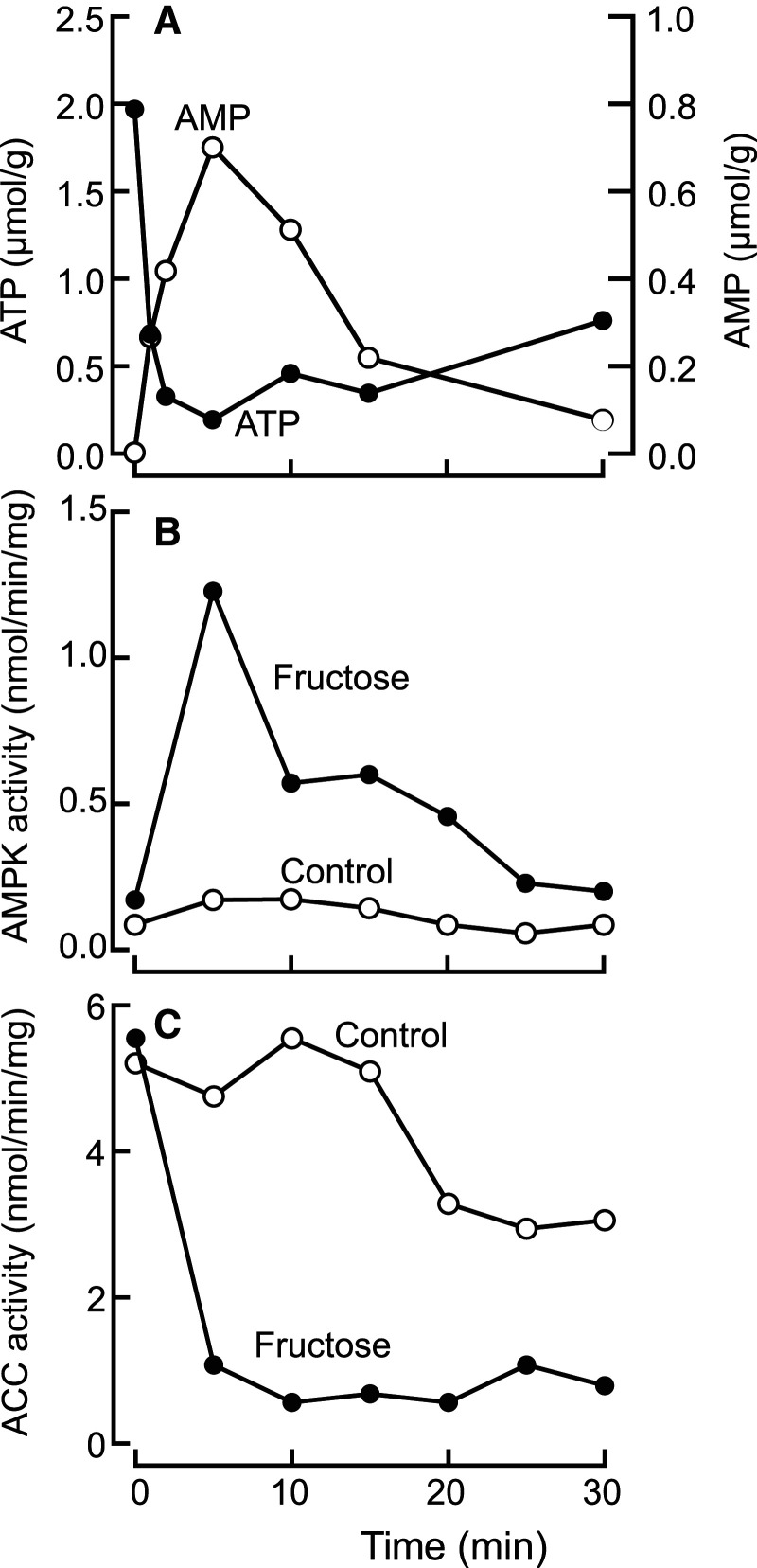
Effects of incubation of isolated rat hepatocytes with fructose. Incubation with 20 mM fructose caused transient decreases in ATP (10-fold) and increases in AMP (>100-fold) (**A**), which were mirrored by a transient increase in the activity of AMPK (**B**) and a decrease in the activity of acetyl-CoA carboxylase (**C**). The acetyl-CoA carboxylase activity started to recover at 40–60 min (not shown). Results redrawn from [53].

Thus, AMPK is a protein kinase that senses cellular energy status. In 1991, my PhD student Frances Moore made an important further observation relating to this. We were looking for ways of examining the physiological role of regulation by AMP, and I came across reports [51,52] that incubation of hepatocytes with high concentrations of fructose transiently depleted ATP due to its rapid conversion by fructokinase to fructose-1-phosphate, which is then more slowly further metabolized via triose phosphates. Sure enough, Frances found that incubation of rat hepatocytes with 20 mM fructose caused a large transient decrease (10-fold) in ATP mirrored by an even larger increase (>100-fold) in AMP [53], as would be expected if the adenylate kinase reaction was close to equilibrium (Figure 3). This was also mirrored by transient increases in AMPK activity and decreases in ACC activity — the latter took longer to recover than AMPK but did do so at later time points not shown in Figure 3. While the decrease in ACC was to be expected, the increase in AMPK was not, because we had assayed the kinase in a resuspended polyethylene glycol precipitate in the presence of saturating AMP, so that any allosteric activation that occurred in the intact cells would have been lost. If we further purified the activated AMPK in the presence of fluoride, then removed the fluoride and treated with protein phosphatase, AMPK became inactivated again. We thus argued that ‘the simplest explanation of these results was that AMP promotes a conformational change in the kinase subunit, which as well as activating directly, makes it a better substrate for the kinase kinase, or a worse substrate for the kinase phosphatase' [53]. In the same paper we provided evidence that AMP binding did promote phosphorylation by an endogenous ‘kinase kinase', which was confirmed much later with more highly purified components [54]. My PhD student Steve Davies also later provided convincing evidence that AMP binding to AMPK inhibited Thr172 dephosphorylation by protein phosphatases [55]. Thus, AMP activated AMPK by three effects rather than one, making the system exquisitely sensitive [56].

The finding that AMP promoted phosphorylation and activation of AMPK made it imperative to define the kinase(s) upstream of AMPK which, despite being first observed by Tom Ingebritsen in 1978 [14], had still not been identified. A new PhD student, Simon Hawley, started in 1992 and I set him the task of purifying the upstream kinase. Simon managed to purify it 1000-fold from rat liver, and with the help of Dave Carling and Matthew Davison at Zeneca, we showed that it phosphorylated Thr172 on the α subunit of AMPK [57]. Thr172 was located in the middle of the ‘activation loop' within which many protein kinases are activated by phosphorylation [58]. Identification of Thr172 also allowed the development of phosphospecific antibodies against that site [59] — the majority of researchers that work on AMPK today do not monitor its kinase activity directly, but instead simply probe Western blots with anti-pT172 antibodies.

It turned out later that rat liver was not the best source for the upstream kinase (for example, it is much more abundant in testis [60]), but our assay did not work well in crude extracts so we could not easily do a survey to find the richest source. Despite much effort, Simon could not identify the upstream kinase in his purified preparations from rat liver — at one point we identified a protein kinase within his fractions, but it turned out to be another ‘red herring'. In the year 2000 a new postdoc, Catherine Sutherland, started — since she had worked on yeast for her PhD in Australia, I put her on identifying the upstream kinase acting on the SNF1 complex, the AMPK orthologue in *S. cerevisiae* — we were assisted by a Dundee colleague who was a yeast biologist, Mike Stark. My PhD student Wayne Wilson had already shown that the upstream kinase purified from rat liver by Simon Hawley would activate the yeast SNF1 complex [61]. The yeast genome had recently been completed, and it encoded 119 protein kinases, at least one of which must be the elusive upstream kinase. We obtained from Mike Snyder at Yale a library of yeast strains each of which expressed a single yeast kinase as a GST fusion — Catherine purified all 119 on glutathione-Sepharose and tested them for their ability to activate dephosphorylated AMPK. Remarkably, she got just one hit, Elm1. I must confess that I sat on this result for a while, not knowing quite what to make of it. One problem was that the phenotype of *elm1* mutants was already known (ELM stands for ELongated Morphology), and it was not really what one would expect for a kinase acting upstream of the SNF1 complex. Another was that some of the GST:Elm1 preparations that Catherine made failed to activate AMPK, and we were in fact very fortunate that the first one she made did work, otherwise we might have missed it altogether! Catherine eventually worked out that those GST-Elm1 preparations that did work in our assay were truncated, with a C-terminal auto-inhibitory domain having been removed by proteolytic degradation. She went on to show that expression of the truncated, activated form of Elm1 caused an elongated morphology and a pseudohyphal growth form caused by a failure of new cell buds to separate from their parent; strangely, this was quite similar to the phenotype of a null mutant. She was then able to show that the phenotype caused by expression of the activated form, although not that of the null mutant, was abolished in the absence of Snf1, providing genetic evidence that Elm1 did indeed act upstream of Snf1. I started preparing a manuscript but, just before submission, I got an e-mail from Martin Schmidt in Pittsburgh (whom I had never met, but whose previous work on the SNF1 complex I knew of) saying that he had a draft manuscript describing kinases upstream of Snf1 in yeast and would like my thoughts on this. I replied that I would love to see his manuscript, but that I had one as well! Rather dramatically, we agreed to exchange manuscripts by e-mail at 1 pm UK time the next day. I sent off mine at 1 pm and waited rather anxiously — I was very relieved to get his manuscript an hour later (probably something to do with the UK and the US not switching to Summer/Daylight saving time on the same weekend)! I was delighted to see that Martin had identified two potential upstream kinases that were different from Elm1, i.e. Pak1 (now renamed Sak1) and Tos3. A knockout of both genes by Martin had failed to generate a *snf1-*like phenotype, i.e. failure to grow on carbon sources other than glucose, which might have been expected of genes encoding kinases upstream of Snf1. Excitingly, however, the kinase domains of Sak1, Tos3 and Elm1 were very closely related in sequence and lay on their own little branch of the yeast kinase family tree. Martin was quickly able to generate a triple null mutant of Elm1, Sak1 and Tos3, and it had a beautiful *snf1-like* phenotype that was rescued by expression from a plasmid of any one of the three. Thus, there were three upstream kinases in yeast that acted redundantly, explaining why they had not emerged from the original genetic screen that had yielded the *snf1* and *snf4* mutants. Martin and our group then published a joint paper [62], while Marian Carlson, working with Dave Carling, published similar findings almost simultaneously [63].

Of course, what I was really interested in was the equivalent upstream kinase in humans. As soon as I heard that Martin had got the exciting results with the triple mutant I looked up the three upstream kinases in the *S. cerevisiase* genome database, where the entry for each gene had a section listing potential mammalian orthologues. These all mentioned *STK11*, a protein kinase gene I had not heard of. However, when I searched PubMed with *STK11* it came up, to my great surprise, with over 100 papers. This was my ‘eureka' moment when I suddenly realized that *STK11* was the gene name for the protein kinase LKB1, which my colleague Dario Alessi had been working on in Dundee for 3 or 4 years. The person in the whole world best qualified to help me was therefore just down the corridor! With mounting excitement, I went to see Dario that same day and he was equally enthusiastic. He had started working on LKB1 because it was a protein kinase in which loss-of-function mutations caused an inherited predisposition to cancer in humans called Peutz–Jeghers syndrome. Dario had worked out quite a bit about LKB1, including that it was only active as a heterotrimeric complex with two accessory subunits, STRAD and MO25 [64], but he had no idea what its real downstream target(s) were. I, on the other hand, had a downstream target (Thr172 on AMPK) for which I hoped that LKB1 was the elusive upstream kinase, so each of us potentially had the answer to the other's greatest problem.

**Figure 4.**
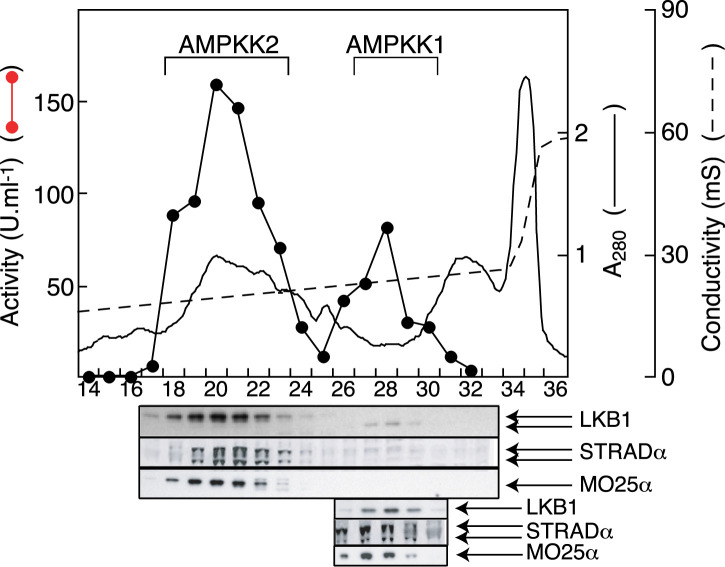
Separation of two AMP-activated protein kinase kinase (AMPKK) activities by chromatography on Q-Sepharose, and their correspondence with LKB1. While experimenting with different elution conditions for the Q-Sepharose step of our published purification protocol for AMPKK [57], Simon Hawley found that he could resolve two peaks of kinase kinase activity (upper graph, filled circles). By blotting fractions with antibodies generated by Dario Alessi's group, we showed that both corresponded with peaks of LKB1, STRAD-α and MO25-α. The lower set of blots were of the same fractions but using a higher protein loading. Careful inspection and additional blots revealed that the LKB1 polypeptide in AMPKK1 migrated faster than that in AMPKK2 — it was shown later to be an alternately spliced variant with a different C-terminus [60]. STRAD-α was always detected as a doublet, for reasons that remain unclear. Results redrawn from [66].

It was by now quite late in the day, but we agreed to do an experiment the following morning. Simon Hawley did a two stage assay to test the ability of a recombinant LKB1:STRAD:MO25 complex made by Dario's group to activate the AMPK complex. This worked so well that we knew we had the right answer even before putting the filter papers into the scintillation counter, just by holding them up to a Geiger counter! However, we became aware that Dave Carling was on to this as well, so there was a rush to publication. A frenzied few weeks followed, in which we used antibodies raised by Dario to show that two upstream kinases purified from rat liver by Simon were indeed both LKB1:STRAD:MO25 complexes (Figure 4), that recombinant LKB1:STRAD:MO25 complexes expressed by Dario's group activated AMPK by phosphorylating Thr172, and that activation of AMPK by energy stress in LKB1-null MEFs or in HeLa cells (a famous human cancer cell line that carries a large deletion in the *STK11* gene [65]) was defective, but could be rescued by re-expressing LKB1.

Dario and I had recently had a visit from an editor of the Journal of Biology, a new journal whose aim was to compete with Nature and Science while providing free colour Figures and free Open Access. Dario and I particularly liked the latter idea, so agreed to submit our paper there. It received very tough but fair reviews that requested additional experiments, but the editorial staff did a great job in producing the final revised version as rapidly as they could. The resulting paper [66] remains by far the most highly cited paper in the Journal of Biology and presumably always will be, because the journal (which eventually became BMC Biology) no longer exists! In the meantime, Dave Carling and Marian Carlson published quite similar findings [67], as did Reuben Shaw, then a postdoc in Lew Cantley's laboratory, a few months later [68].

The identification of other upstream kinases that phosphorylated Thr172 and activated AMPK soon followed, including the Ca^2+^/calmodulin-dependent protein kinase CaMKK2 [69–71] and the cytokine-activated kinase TAK1 [72–74]. However, the discovery that LKB1 was the principal upstream kinase that phosphorylates and activates AMPK during energy stress remains the most exciting moment of my research career because it connected a kinase known for regulation of metabolism (AMPK) with one known to have a role in cancer, two fields that previously had not seemed to have had much connection. As stated above, the *STK11* gene that encoded LKB1 had been discovered as the gene mutated in Peutz–Jeghers syndrome [75,76]. Humans with this syndrome inherit heterozygous loss-of-function mutations in this gene and develop numerous benign growths (hamartomas) in the intestine that require frequent surgery. However, they also have a greatly increased risk of developing malignant tumours at many different sites and tend to die from cancer at a relatively young age. In addition, somatic mutations in *STK11* are found in many spontaneous (i.e. non-inherited) cancers, especially in the commonest form of lung cancer, adenocarcinoma, where they occur in up to 20% of all cases [77–79]. LKB1 is thus a classical tumour suppressor, which raised the intriguing question of whether AMPK was exerting the tumour suppressor effects of LKB1. A caveat emerged when Dario's group showed that LKB1, in addition to AMPK, also phosphorylated and activated 12 members of the AMPK-related kinase (ARK) family that have kinase domains related to AMPK [80] — any one of these could potentially exert the tumour suppressor effects of LKB1. Nevertheless, AMPK is the only member of the ARK family known to inhibit most biosynthetic pathways (i.e. cell growth) as well as progress through the cell cycle (i.e. cell division) [81], and therefore still seemed the best candidate to account for the tumour suppressor effects of LKB1.

## The 2010s to the present day — AMPK as a double-edged sword in cancer

Although I was keen to test the idea that AMPK was a tumour suppressor, getting into a position to do this required overcoming a certain degree of inertia. This was partly because I had no real experience or track record of research in cancer, so I felt that I might lack credibility with the relevant funding bodies. In addition, cancer is a disorder involving interactions between many different cell types, and to study it experimentally really required the use of genetically modified mice, another area in which I lacked expertise. Eventually, after an initial application was turned down, Cancer Research UK agreed to fund a Programme grant aimed at testing the idea that AMPK might exert the tumour suppressor effects of LKB1.

**Figure 5.**
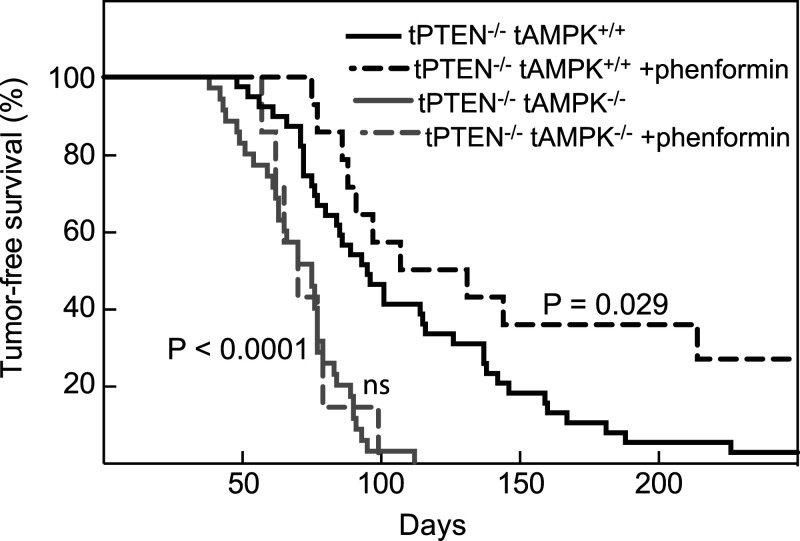
Tumour-free survival curves for mice with T cell-specific knockout of PTEN (PTEN^−/−^) with or without T cell-specific knockout of AMPK-α1 (tAMPK^−/−^), treated with or without phenformin. Phenformin was administered in the drinking water from the time of weaning at about 28 days (which was well before any lymphomas became evident). AMPK knockout caused earlier onset and more rapid development of lymphomas (*P* < 0.0001); phenformin treatment caused later onset and slower development of lymphomas (*P* = 0.029) but only if AMPK was present in the developing T cells (tAMPK^+/+^). Phenformin had no effect (ns) in AMPK knockout T cells (tAMPK^−/−^). Results from [85].

My original idea was to study a mouse model of lung cancer, which had been used previously to study effects of LKB1 loss, in which an oncogenic mutant of K-Ras was switched on, with or without knockout of LKB1, by administration to the lungs via nasal inhalation of a viral vector expressing Cre recombinase [78]. One problem with this, as pointed out by some reviewers on my first Cancer Research UK application, was that the tumour progenitor cells were likely to express both the α1 and α2 isoforms of AMPK, so the project would require combining five mutant alleles by mouse breeding (one lox-STOP-lox allele to switch on the K-Ras oncogene, plus two floxed alleles for each of the α1 and α2 genes to knock them out). While other groups did eventually get this to work (albeit with rather conflicting results) [82,83], it was perhaps a little over-ambitious for a novice at the game like me. Fortunately, my colleague Doreen Cantrell suggested an easier alternative, a mouse model of T-ALL (T cell acute lymphoblastic leukaemia/lymphoma), which required combining T cell-specific knockouts of the tumour suppressor PTEN and AMPK. This model had the huge advantage that Doreen had shown that developing T cells in the thymus express only the α1 isoform of AMPK [84], so that it was not necessary to knock out α2 as well. In addition, Doreen could provide practical help and advice, and already had the required mouse strains. A Wellcome Trust Clinical PhD student, Madhu Dandapani, obtained some promising initial results and then a Spanish postdoc, Diana Vara-Ciruelos, took over. Diana found that if T cell-specific knockout of PTEN was combined with knockout of AMPK the tumours arose earlier and caused a faster development of disease than those with PTEN loss alone (Figure 5), providing genetic evidence that AMPK was indeed acting as a tumour suppressor [85]. These results confirmed that basal AMPK was delaying tumourigenesis, but we were also interested in whether activating AMPK would delay it even further. We were particularly keen to test the effects of the AMPK-activating biguanide drug metformin [86] which, following our identification of LKB1 as the upstream kinase for AMPK (a known target for the drug), had been shown by Andrew Morris (then Professor of Diabetic Medicine in Dundee) to be associated with a reduced risk of cancer when used to treat type 2 diabetes in humans [87]. Disappointingly, treatment of our mice with oral metformin from the time of weaning (which was well before any lymphomas became evident) had no effect on the occurrence of T-ALL. However, we showed that this was likely to be because metformin (which requires transporters of the SLC22 family, such as OCT1, to get into cells) failed to enter the thymus and activate AMPK. In contrast, treatment with the more hydrophobic biguanide phenformin, which did enter the thymus and activate AMPK, delayed the onset of T-ALL b*ut only if AMPK was present in the developing T cells* (Figure 5). Since this model used T cell-specific knockouts, where expression of PTEN and AMPK would have been normal in all other cells, this proved that pharmacological activation of AMPK could protect against tumour development via a cell-autonomous effect occurring within the tumour progenitor cells themselves [85].

Disturbingly, while this project was in progress a paper came out from Jeff Rathmell's group on the effect of AMPK knockout in another mouse model of T-ALL, which came to completely opposite conclusions [88]. However, the model they used was quite different to ours. Firstly, haematopoietic progenitor cells carrying floxed alleles of the AMPK-α1 gene *(Prkaa1)* and a tamoxifen-inducible Cre recombinase gene had been transformed *in vitro* by expressing an oncogenic Notch mutant. The cells were then amplified and used to inoculate mice that had been irradiated to repress their own immune systems. After 10 days to allow T-ALL to become established, the mice were treated with or without tamoxifen to induce Cre recombinase and hence knock out AMPK. In this model, AMPK knockout reduced the number of T-ALL cells recovered and enhanced survival of the mice, suggesting that the presence of AMPK-α1 was acting as a tumour promoter. How could these results [88] be reconciled with our own [85]? The key difference, which I believe to be crucial, is that we had knocked out AMPK *before* T-ALL had arisen, whereas Rathmell's group had knocked it out only *after* disease had become established. Before cancer has arisen, AMPK may act as a tumour suppressor that protects against the development of cancer by restraining the metabolic changes required for rapid cell growth. Once cancer has arisen, however (despite the best efforts of AMPK to prevent it!) AMPK switches to become a tumour promoter, most likely because it protects tumour cells against energetic stress and other type of stress caused by their rapid growth, and therefore increases their survival.

These results were intriguing, but what about human cancer? The role of specific genes in human cancer can be investigated by interrogating the numerous cancer genome studies using the cBioPortal database (http://www.cbioportal.org/ [89,90]). As an example, when we searched the database for genetic changes in the *STK11* gene (encoding LKB1) in human cancers, it was found to be quite frequently mutated or deleted, particularly in lung adenocarcinoma where this occurred in 15–20% of cases (as indeed had already been reported [77,78]). This is exactly what would be expected for a tumour suppressor. Inspired by initial observations made by Daniel Murphy and co-workers [91], we searched the database to look for genetic changes in cancer for all seven AMPK genes [79]. Interestingly, the *PRKAA2* gene encoding AMPK-α2 displays mis-sense mutations in 10–20% of cases of certain cancers (for reasons that remain unclear, this was particularly common in skin cancer and melanoma). This behaviour might be expected for a tumour suppressor. However, the *PRKAA1* gene, encoding AMPK-α1, displayed exactly the opposite behaviour in that it was *amplified* in many cancers, which is what one might expect for a tumour promoter. Amplification of *PRKAA1* was particularly common in lung adenocarcinoma, where it occurred in around 10% of cases. It was particularly intriguing that this cancer type, in which the gene encoding the downstream kinase, AMPK-α1, was often amplified, was also the type where the gene encoding the upstream kinase, LKB1, was most frequently mutated or deleted — at first sight, this might seem paradoxical.

Amplification of genes usually involves whole segments of chromosome rather than single genes, so one possibility was that *PRKAA1* was adjacent to an oncogene whose amplification was being selected for, with *PRKAA1* coming along for the ride as an innocent passenger. However, this became a less likely explanation when further analysis showed that mutation or deletion of *STK11* and amplification of *PRKAA1* tended to be mutually exclusive. For example, in 230 cases of lung adenocarcinoma in The Cancer Genome Atlas [92], *STK11* was either deleted or mutated in 43 (19%) and *PRKAA1* was amplified in 22 (10%). However, these changes never coincided, which would be expected (*P* < 0.005) if they were happening independently. After all, there would not be much point in amplifying the expression of AMPK-α1, if that was being actively selected for, if functional LKB1 was not there to activate it. Like many other cancers, the most frequent mutations (47% of cases) in this study were in the *TP53* gene, encoding the tumour suppressor p53 [92]. Intriguingly, amplification of *PRKAA1* almost always co-occurred with mutations in *TP53*, which once again would not be expected (*P* < 0.001) if these changes were occurring independently.

Why might amplification of AMPK-α1 be selected for in cancers that carry *TP53* mutations? The protein p53 is a transcription factor, and many of the mutations found in cancer interfere with its transcriptional activity. p53 normally has a very short half-life, but it becomes stabilized and/or activated in response to DNA damage [93]. It then causes a G1 cell cycle arrest, in part by inducing transcription of genes such as *CDKN1A* encoding the inhibitor of G1 cyclin-dependent kinases, p21^CIP1^, thus allowing time for DNA damage to be repaired. Alternatively, if the DNA damage is severe, p53 may induce cell death via senescence or apoptosis [93]. Tumour cells lacking functional p53 are therefore susceptible to agents that cause DNA damage (many of which are used in cancer treatment), and multiple genetic alterations can accumulate in p53 mutant cancers.

Our current hypothesis is that increased expression of AMPK-α1 is selected for in *TP53* mutant tumours because it can partially compensate for lack of p53 function. In 2008, Fu et al. [94] reported that the anti-cancer agent etoposide, which causes double-strand breaks in DNA by inhibiting topoisomerase II [95], activated AMPK; other DNA-damaging agents used in cancer treatment, such as ionizing radiation [96], or the ribonuclease inhibitor hydroxurea [97] have also been reported to do the same. Diana Vara-Ciruelos and Madhu Dandapani in my laboratory confirmed that etoposide treatment of cells activated AMPK, and also showed that cells with a double knockout of AMPK-α1 and -α2 were more sensitive to cell death induced by etoposide [98]. This may in part be because AMPK activation causes a cell cycle arrest in G1 phase [81], allowing more time for DNA repair before entry into S phase, during which cells are much more sensitive to DNA-damaging agents such as etoposide. Moreover, it was subsequently shown that AMPK may be directly involved in the repair of DNA damage via phosphorylation of various targets, including EXO1, an exonuclease that plays a key role in DNA repair by homologous recombination [97], 53BP1, which plays a key role in DNA repair by non-homologous end joining [99], and others [100,101]. Other interesting findings made by Diana and Madhu were that activation of AMPK by etoposide only occurred within the nucleus and was completely specific for AMPK complexes containing the α1 and not the α2 isoform [98]. In addition, activation by etoposide did not require LKB1 and was mediated by the alternate upstream kinase CaMKK2, apparently triggered by an increase in nuclear Ca^2+^, as also shown by Li et al. [97]; the exact source of this Ca^2+^ remains unclear at present.

This activation of AMPK in response to DNA damage and its involvement in key mechanisms of DNA repair may help to explain why amplification of AMPK occurs in some cancers with loss-of-function mutations in *TP53*, since overexpression of AMPK may be able to compensate for the increased sensitivity of p53-null tumours to DNA damage, thus increasing survival of the tumour cells. Under these circumstances, AMPK is effectively acting as a tumour promoter. However, two key questions remained: (i) why did activation of AMPK in response to DNA damage only occur within the nucleus? and (ii) why is it only the *PRKAA1* gene (encoding the α1 isoform) that is amplified in some cancers? Potential answers to both questions emerged when I was contacted, with a view to a collaboration, by Sehamuddin Galadari at New York University in Abu Dhabi. His postdoc Anees Cheratta had found that AMPK-α1 was cleaved by the so-called ‘executioner' proteinase, caspase-3, during the early stages of apoptosis induced by DNA damage. They mapped the cleavage site to Asp-529 [102], which removes just 30 residues from the C-terminal end (which may explain why the cleavage was missed during previous studies of DNA damage, particularly since only a small proportion of total AMPK-α1 may be affected). The cleavage site (which lies within a flexible region of the α subunit called the ST loop [103]) removes the final α-helix of the α subunit, which contains a well-characterised nuclear export sequence (NES) [104]. Both the α1 and α2 isoforms contain this NES and appear to shuttle between the nucleus and the cytoplasm, although the locations of any nuclear localization sequences remain unclear. However, if α1 has been cleaved by caspase-3 it has lost its NES and will become trapped in the nucleus, where it may become activated by CaMKK2 due to the rising levels of nuclear Ca^2+^. Note that Asp529 and the surrounding sequence, which fits the consensus motif for caspase-3 cleavage rather well, appears to be conserved across all vertebrate α1 sequences but that an aspartate is not present at that position in any vertebrate α2 sequences. Our contribution to the resulting paper [102] was to confirm that the caspase-3 cleavage, when conducted in cell-free assays, did not affect the stability of existing α1βγ complexes, nor did it affect their ability to be allosterically activated by AMP or to be activated by phosphorylation at Thr172 by upstream kinases including CaMKK2. Thus, AMPK complexes containing α1 may have a specialized role in nuclear functions, such as the response to DNA damage. Under these circumstances AMPK-α1 may act as a tumour promoter by compensating for lack of p53 and protecting tumour cells against cell death caused by DNA damage. Thus, AMPK is a ‘double-edged sword', initially acting as a tumour suppressor that protects against cancer but then, once cancer has arisen due to gain/loss-of-function mutations in other oncogenes/tumour suppressors, switching to become a promoter of tumour growth, most likely by protecting cancer cells against genotoxic and other stresses that might otherwise cause cell death. Genotoxic stress might be generated, for example, by cytotoxic drugs used in cancer treatment and, if our hypothesis is correct, inhibitors of AMPK might turn out to be a useful adjunct to such treatments.

## Conclusions

The pursuit of what turned out to be AMPK, which started in that bar in Dundee the evening I went down there with Philip Cohen in 1976, has become a life-long obsession that has kept my research career going for over 46 years. In 2022, an average of ≈60 publications that mention AMPK appear every week, which makes it increasingly difficult to keep up with my own field! One very pleasant way to try to overcome this problem is to attend the international conferences on AMPK that were started by Neil Ruderman in 2000 and have been held every 2 years ever since (with one gap because of Covid-19), or the smaller European workshops started by Dietbert Neumann that have latterly been held in alternate years. The AMPK research community is now a large one, which I believe is generally co-operative and welcoming, and it has been a privilege for me to be part of that community.

## Open Access

Open access for this article was enabled by the participation of University of Dundee in an all-inclusive *Read & Publish* agreement with Portland Press and the Biochemical Society under a transformative agreement with JISC.

## Abbreviations

ACC: acetyl-CoA carboxylase
AMPK: AMP-activated protein kinase
ARK: AMPK-related kinase
HMGCR: HMG-CoA reductase
NES: nuclear export sequence
PKA: protein kinase
T-ALL: T cell acute lymphoblastic leukaemia/lymphoma

## References

[1] Hardie, D.G., Matthews, H.R. and Bradbury, E.M. (1976) Cell-cycle dependence of two nuclear histone kinase enzyme activities. Eur. J. Biochem. 66, 37–42 10.1111/j.1432-1033.1976.tb10422.x 182490

[2] Inglis, R.J., Langan, T.A., Matthews, H.R., Hardie, D.G. and Bradbury, E.M. (1976) Advance of mitosis by histone phosphokinase. Exp. Cell Res. 97, 418–425 10.1016/0014-4827(76)90634-0 174923

[3] Nurse, P. (1990) Universal control mechanism regulating onset of M-phase. Nature 344, 503–508 10.1038/344503a0 2138713

[4] Hardie, D.G. and Stansfield, D.A. (1977) Endogenous phosphorylation of microsomal proteins in bovine corpus luteum. Tenfold activation by adenosine 3':5'-cyclic monophosphate. Biochem. J. 164, 213–221 10.1042/bj1640213 195580 PMC1164776

[5] Carlson, C.A. and Kim, K.H. (1973) Regulation of hepatic acetyl coenzyme A carboxylase by phosphorylation and dephosphorylation. J. Biol. Chem. 248, 378–380 10.1016/S0021-9258(19)44486-4 4692841

[6] Hardie, D.G. and Cohen, P. (1978) The regulation of fatty acid biosynthesis: simple procedure for the purification of acetyl CoA carboxylase from lactating rabbit mammary gland, and its phosphorylation by endogenous cyclic AMP-dependent and -independent protein kinase activities. FEBS Lett. 91, 1–7 10.1016/0014-5793(78)80005-2 27383

[7] Brownsey, R.W. and Hardie, D.G. (1980) Regulation of acetyl-CoA carboxylase: identity of sites phosphorylated in intact cells treated with adrenaline and in vitro by cyclic AMP-dependent protein kinase. FEBS Lett. 120, 67–70 10.1016/0014-5793(80)81048-9 6108241

[8] Holland, R., Witters, L.A. and Hardie, D.G. (1984) Glucagon inhibits fatty acid synthesis in isolated hepatocytes via phosphorylation of acetyl-CoA carboxylase by cyclic-AMP-dependent protein kinase. Eur. J. Biochem. 140, 325–333 10.1111/j.1432-1033.1984.tb08105.x 6143665

[9] Sim, A.T.R. and Hardie, D.G. (1988) The low activity of acetyl-CoA carboxylase in basal and glucagon-stimulated hepatocytes is due to phosphorylation by the AMP-activated protein kinase and not cyclic AMP-dependent protein kinase. FEBS Lett. 233, 294–298 10.1016/0014-5793(88)80445-9 2898386

[10] Hardie, D.G. and Cohen, P. (1979) Dephosphorylation and activation of acetyl-CoA carboxylase from lactating rabbit mammary gland. FEBS Lett. 103, 333–338 10.1016/0014-5793(79)81356-3 38145

[11] Hardie, D.G. and Guy, P.S. (1980) Reversible phosphorylation and inactivation of acetyl-CoA carboxylase from lactating rat mammary gland by cyclic AMP-dependent protein kinase. Eur. J. Biochem. 110, 167–177 10.1111/j.1432-1033.1980.tb04852.x 6108209

[12] Campbell, D.G., Hardie, D.G. and Vulliet, P.R. (1986) Identification of four phosphorylation sites in the N-terminal region of tyrosine hydroxylase. J. Biol. Chem. 261, 10489–10492 10.1016/S0021-9258(18)67410-1 2874140

[13] Beg, Z.H., Allmann, D.W. and Gibson, D.M. (1973) Modulation of 3-hydroxy-3-methylglutaryl coenzyme: a reductase activity with cAMP and with protein fractions of rat liver cytosol. Biochem. Biophys. Res. Commun. 54, 1362–1369 10.1016/0006-291X(73)91137-6 4356818

[14] Ingebritsen, T.S., Lee, H.S., Parker, R.A. and Gibson, D.M. (1978) Reversible modulation of the activities of both liver microsomal hydroxymethylglutaryl coenzyme A reductase and its inactivating enzyme. Evidence for regulation by phosphorylation-dephosphorylation. Biochem. Biophys. Res. Commun. 81, 1268–1277 10.1016/0006-291X(78)91273-1 666819

[15] Yeh, L.A., Lee, K.H. and Kim, K.H. (1980) Regulation of rat liver acetyl-CoA carboxylase. Regulation of phosphorylation and inactivation of acetyl-CoA carboxylase by the adenylate energy charge. J. Biol. Chem. 255, 2308–2314 10.1016/S0021-9258(19)85891-X 6102090

[16] Ferrer, A., Caelles, C., Massot, N. and Hegardt, F.G. (1985) Activation of rat liver cytosolic 3-hydroxy-3-methylglutaryl coenzyme A reductase kinase by adenosine 5'-monophosphate. Biochem. Biophys. Res. Commun. 132, 497–504 10.1016/0006-291X(85)91161-1 4062938

[17] Chin, D.J., Luskey, K.L., Anderson, R.G.W., Faust, J.R., Goldstein, J.L. and Brown, M.S. (1982) Appearance of crystalloid endoplasmic reticulum in compactin resistant Chinese hamster cells with a 500-fold increase in 3-hydroxy-3-methylglutaryl Coenzyme A reductase. Proc. Natl Acad. Sci. U.S.A. 79, 1185–1189 10.1073/pnas.79.4.1185 6951166 PMC345926

[18] Brown, M.S., Goldstein, J.L. and Dietschy, J.M. (1979) Active and inactive forms of 3-hydroxy-3-methylglutaryl Coenzyme A reductase in the liver of the rat. Comparison with the rate of cholesterol synthesis in different physiological states. J. Biol. Chem. 254, 5144–5149 10.1016/S0021-9258(18)50571-8 376507

[19] Brown, M.S., Brunschede, G.Y. and Goldstein, J.L. (1975) Inactivation of 3-hydroxy-3-methylglutaryl coenzyme A reductase *in vitro*. An adenine nucleotide-dependent reaction catalyzed by a factor in human fibroblasts. J. Biol. Chem. 250, 2502–2509 10.1016/S0021-9258(19)41629-3 804475

[20] Carling, D., Zammit, V.A. and Hardie, D.G. (1987) A common bicyclic protein kinase cascade inactivates the regulatory enzymes of fatty acid and cholesterol biosynthesis. FEBS Lett. 223, 217–222 10.1016/0014-5793(87)80292-2 2889619

[21] Carling, D. and Hardie, D.G. (1989) The substrate and sequence specificity of the AMP-activated protein kinase. Phosphorylation of glycogen synthase and phosphorylase kinase. Biochim. Biophys. Acta 1012, 81–86 10.1016/0167-4889(89)90014-1 2567185

[22] Garton, A.J., Campbell, D.G., Carling, D., Hardie, D.G., Colbran, R.J. and Yeaman, S.J. (1989) Phosphorylation of bovine hormone-sensitive lipase by the AMP-activated protein kinase. A possible antilipolytic mechanism. Eur. J. Biochem. 179, 249–254 10.1111/j.1432-1033.1989.tb14548.x 2537200

[23] Munday, M.R., Campbell, D.G., Carling, D. and Hardie, D.G. (1988) Identification by amino acid sequencing of three major regulatory phosphorylation sites on rat acetyl-CoA carboxylase. Eur. J. Biochem. 175, 331–338 10.1111/j.1432-1033.1988.tb14201.x 2900138

[24] Lopez-Casillas, F., Bai, D.H., Luo, X.C., Kong, I.S., Hermodson, M.A. and Kim, K.H. (1988) Structure of the coding sequence and primary amino acid sequence of acetyl-coenzyme A carboxylase. Proc. Natl Acad. Sci. U.S.A. 85, 5784–5788 10.1073/pnas.85.16.5784 2901088 PMC281849

[25] Davies, S.P., Sim, A.T. and Hardie, D.G. (1990) Location and function of three sites phosphorylated on rat acetyl-CoA carboxylase by the AMP-activated protein kinase. Eur. J. Biochem. 187, 183–190 10.1111/j.1432-1033.1990.tb15293.x 1967580

[26] Davies, S.P., Carling, D. and Hardie, D.G. (1989) Tissue distribution of the AMP-activated protein kinase, and lack of activation by cyclic-AMP-dependent protein kinase, studied using a specific and sensitive peptide assay. Eur. J. Biochem. 186, 123–128 10.1111/j.1432-1033.1989.tb15185.x 2574667

[27] Cool, B., Zinker, B., Chiou, W., Kifle, L., Cao, N., Perham, M. et al. (2006) Identification and characterization of a small molecule AMPK activator that treats key components of type 2 diabetes and the metabolic syndrome. Cell Metab. 3, 403–416 10.1016/j.cmet.2006.05.005 16753576

[28] Myers, R.W., Guan, H.P., Ehrhart, J., Petrov, A., Prahalada, S., Tozzo, E. et al. (2017) Systemic pan-AMPK activator MK-8722 improves glucose homeostasis but induces cardiac hypertrophy. Science 357, 507–511 10.1126/science.aah5582 28705990

[29] Cokorinos, E.C., Delmore, J., Reyes, A.R., Albuquerque, B., Kjobsted, R., Jorgensen, N.O. et al. (2017) Activation of skeletal muscle AMPK promotes glucose disposal and glucose lowering in non-human primates and mice. Cell Metab. 25, 1147–1159 10.1016/j.cmet.2017.04.010 28467931

[30] Weekes, J., Ball, K.L., Caudwell, F.B. and Hardie, D.G. (1993) Specificity determinants for the AMP-activated protein kinase and its plant homologue analysed using synthetic peptides. FEBS Lett. 334, 335–339 10.1016/0014-5793(93)80706-Z 7902296

[31] Dale, S., Wilson, W.A., Edelman, A.M. and Hardie, D.G. (1995) Similar substrate recognition motifs for mammalian AMP-activated protein kinase, higher plant HMG-CoA reductase kinase-A, yeast SNF1, and mammalian calmodulin-dependent protein kinase I. FEBS Lett. 361, 191–195 10.1016/0014-5793(95)00172-6 7698321

[32] Clarke, P.R. and Hardie, D.G. (1990) Regulation of HMG-CoA reductase: identification of the site phosphorylated by the AMP-activated protein kinase in vitro and in intact rat liver. EMBO J. 9, 2439–2446 10.1002/j.1460-2075.1990.tb07420.x 2369897 PMC552270

[33] Steinberg, G.R. and Hardie, D.G. (2022) New insights into activation and function of the AMP-activated protein kinase (AMPK). Nat. Rev. Cell Mol. Biol. 10.1038/s41580-022-00547-x 36316383

[34] Hawley, S.A., Gadalla, A.E., Olsen, G.S. and Hardie, D.G. (2002) The antidiabetic drug metformin activates the AMP-activated protein kinase cascade via an adenine nucleotide-independent mechanism. Diabetes 51, 2420–2425 10.2337/diabetes.51.8.2420 12145153

[35] Davies, S.P., Hawley, S.A., Woods, A., Carling, D., Haystead, T.A. and Hardie, D.G. (1994) Purification of the AMP-activated protein kinase on ATP-gamma-sepharose and analysis of its subunit structure. Eur. J. Biochem. 223, 351–357 10.1111/j.1432-1033.1994.tb19001.x 8055903

[36] Mitchelhill, K.I., Stapleton, D., Gao, G., House, C., Michell, B., Katsis, F. et al. (1994) Mammalian AMP-activated protein kinase shares structural and functional homology with the catalytic domain of yeast Snf1 protein kinase. J. Biol. Chem. 269, 2361–2364 10.1016/S0021-9258(17)41951-X 7905477

[37] Carling, D., Clarke, P.R., Zammit, V.A. and Hardie, D.G. (1989) Purification and characterization of the AMP-activated protein kinase. Copurification of acetyl-CoA carboxylase kinase and 3-hydroxy-3-methylglutaryl-CoA reductase kinase activities. Eur. J. Biochem. 186, 129–136 10.1111/j.1432-1033.1989.tb15186.x 2598924

[38] Carling, D., Aguan, K., Woods, A., Verhoeven, A.J.M., Beri, R.K., Brennan, C.H. et al. (1994) Mammalian AMP-activated protein kinase is homologous to yeast and plant protein kinases involved in the regulation of carbon metabolism. J. Biol. Chem. 269, 11442–11448 10.1016/S0021-9258(19)78143-5 7908907

[39] Celenza, J.L. and Carlson, M. (1986) A yeast gene that is essential for release from glucose repression encodes a protein kinase. Science 233, 1175–1180 10.1126/science.3526554 3526554

[40] Stapleton, D., Gao, G., Michell, B.J., Widmer, J., Mitchelhill, K., Teh, T. et al. (1994) Mammalian 5'-AMP-activated protein kinase non-catalytic subunits are homologs of proteins that interact with yeast Snf1 protein kinase. J. Biol. Chem. 269, 29343–29346 10.1016/S0021-9258(18)43879-3 7961907

[41] Celenza, J.L., Eng, F.J. and Carlson, M. (1989) Molecular analysis of the SNF4 gene of *Saccharomyces cerevisiae*: evidence for physical association of the SNF4 protein with the SNF1 protein kinase. Mol. Cell. Biol. 9, 5045–5054 10.1128/mcb.9.11.5045-5054.1989 2481228 PMC363656

[42] Fields, S. and Song, O.K. (1989) A novel genetic system to detect protein-protein interactions. Nature 340, 245–246 10.1038/340245a0 2547163

[43] Yang, X., Hubbard, E.J. and Carlson, M. (1992) A protein kinase substrate identified by the two-hybrid system. Science 257, 680–682 10.1126/science.1496382 1496382

[44] Thornton, C., Snowden, M.A. and Carling, D. (1998) Identification of a novel AMP-activated protein kinase beta subunit isoform that is highly expressed in skeletal muscle. J. Biol. Chem. 273, 12443–12450 10.1074/jbc.273.20.12443 9575201

[45] Woods, A., Cheung, P.C., Smith, F.C., Davison, M.D., Scott, J., Beri, R.K. et al. (1996) Characterization of AMP-activated protein kinase beta and gamma subunits. Assembly of the heterotrimeric complex in vitro. J. Biol. Chem. 271, 10282–10290 10.1074/jbc.271.17.10282 8626596

[46] Cheung, P.C.F., Salt, I.P., Davies, S.P., Hardie, D.G. and Carling, D. (2000) Characterization of AMP-activated protein kinase g subunit isoforms and their role in AMP binding. Biochem. J. 346, 659–669 10.1042/bj3460659 10698692 PMC1220898

[47] Gao, G., Fernandez, C.S., Stapleton, D., Auster, A.S., Widmer, J., Dyck, J.R. et al. (1996) Non-catalytic beta- and gamma-subunit isoforms of the 5'-AMP-activated protein kinase. J. Biol. Chem. 271, 8675–8681 10.1074/jbc.271.15.8675 8621499

[48] Hardie, D.G. and Hawley, S.A. (2001) AMP-activated protein kinase: the energy charge hypothesis revisited. BioEssays 23, 1112–1119 10.1002/bies.10009 11746230

[49] Krebs, H. (1964) The Croonian Lecture, 1963. Gluconeogenesis. Proc. R. Soc. Lond. B. Biol. Sci. 159, 545–564 10.1098/rspb.1964.0019 14130854

[50] Atkinson, D.E. (1977) Cellular Energy Metabolism and its Regulation, Academic Press, New York

[51] Van den Berghe, G. (1986) Fructose: metabolism and short-term effects on carbohydrate and purine metabolic pathways. In Prog. Biochem. Pharmacol., vol. 21 (Macdonald, I. and Vrana, A., eds.). pp. 1–32, Karger, Basel (ISSN 0079-6085, PMID ) 3523498

[52] Carabaza, A., Ricart, M.D., Mor, A., Guinovart, J.J. and Ciudad, C.J. (1990) Role of AMP on the activation of glycogen synthase and phosphorylase by adenosine, fructose, and glutamine in rat hepatocytes. J. Biol. Chem. 265, 2724–2732 10.1016/S0021-9258(19)39862-X 2105932

[53] Moore, F., Weekes, J. and Hardie, D.G. (1991) Evidence that AMP triggers phosphorylation as well as direct allosteric activation of rat liver AMP-activated protein kinase. A sensitive mechanism to protect the cell against ATP depletion. Eur. J. Biochem. 199, 691–697 10.1111/j.1432-1033.1991.tb16172.x 1678349

[54] Ross, F.A., Jensen, T.E. and Hardie, D.G. (2016) Differential regulation by AMP and ADP of AMPK complexes containing different gamma subunit isoforms. Biochem. J. 473, 189–199 10.1042/BJ20150910 26542978 PMC4700476

[55] Davies, S.P., Helps, N.R., Cohen, P.T. and Hardie, D.G. (1995) 5'-AMP inhibits dephosphorylation, as well as promoting phosphorylation, of the AMP-activated protein kinase. Studies using bacterially expressed human protein phosphatase-2C alpha and native bovine protein phosphatase-2AC. FEBS Lett. 377, 421–425 10.1016/0014-5793(95)01313-X 8549768

[56] Hardie, D.G., Salt, I.P., Hawley, S.A. and Davies, S.P. (1999) AMP-activated protein kinase: an ultrasensitive system for monitoring cellular energy charge. Biochem. J. 338, 717–722 10.1042/bj3380717 10051444 PMC1220108

[57] Hawley, S.A., Davison, M., Woods, A., Davies, S.P., Beri, R.K., Carling, D. et al. (1996) Characterization of the AMP-activated protein kinase kinase from rat liver and identification of threonine 172 as the major site at which it phosphorylates AMP-activated protein kinase. J. Biol. Chem. 271, 27879–27887 10.1074/jbc.271.44.27879 8910387

[58] Taylor, S.S. and Kornev, A.P. (2011) Protein kinases: evolution of dynamic regulatory proteins. Trends Biochem. Sci. 36, 65–77 10.1016/j.tibs.2010.09.006 20971646 PMC3084033

[59] Pan, D.A. and Hardie, D.G. (2002) A homologue of AMP-activated protein kinase in Drosophila melanogaster is sensitive to AMP and is activated by ATP depletion. Biochem. J. 367, 179–186 10.1042/bj20020703 12093363 PMC1222868

[60] Towler, M.C., Fogarty, S., Hawley, S.A., Pan, D.A., Martin, D., Morrice, N.A. et al. (2008) A novel short splice variant of the tumour suppressor LKB1 is required for spermiogenesis. Biochem. J. 416, 1–14 10.1042/BJ20081447 18774945

[61] Wilson, W.A., Hawley, S.A. and Hardie, D.G. (1996) Glucose repression/derepression in budding yeast: SNF1 protein kinase is activated by phosphorylation under derepressing conditions, and this correlates with a high AMP:ATP ratio. Curr. Biol. 6, 1426–1434 10.1016/S0960-9822(96)00747-6 8939604

[62] Sutherland, C.M., Hawley, S.A., McCartney, R.R., Leech, A., Stark, M.J., Schmidt, M.C. et al. (2003) Elm1p is one of three upstream kinases for the *Saccharomyces cerevisiae* SNF1 complex. Curr. Biol. 13, 1299–1305 10.1016/S0960-9822(03)00459-7 12906789

[63] Hong, S.P., Leiper, F.C., Woods, A., Carling, D. and Carlson, M. (2003) Activation of yeast Snf1 and mammalian AMP-activated protein kinase by upstream kinases. Proc. Natl Acad. Sci. U.S.A. 100, 8839–8843 10.1073/pnas.1533136100 12847291 PMC166400

[64] Boudeau, J., Baas, A.F., Deak, M., Morrice, N.A., Kieloch, A., Schutkowski, M. et al. (2003) MO25a/b interact with STRADa/b enhancing their ability to bind, activate and localize LKB1 in the cytoplasm. EMBO J. 22, 5102–5114 10.1093/emboj/cdg490 14517248 PMC204473

[65] Wingo, S.N., Gallardo, T.D., Akbay, E.A., Liang, M.C., Contreras, C.M., Boren, T. et al. (2009) Somatic LKB1 mutations promote cervical cancer progression. PLoS One 4, e5137 10.1371/journal.pone.0005137 19340305 PMC2660434

[66] Hawley, S.A., Boudeau, J., Reid, J.L., Mustard, K.J., Udd, L., Makela, T.P. et al. (2003) Complexes between the LKB1 tumor suppressor, STRADa/b and MO25a/b are upstream kinases in the AMP-activated protein kinase cascade. J. Biol. 2, 28 10.1186/1475-4924-2-28 14511394 PMC333410

[67] Woods, A., Johnstone, S.R., Dickerson, K., Leiper, F.C., Fryer, L.G., Neumann, D. et al. (2003) LKB1 is the upstream kinase in the AMP-activated protein kinase cascade. Curr. Biol. 13, 2004–2008 10.1016/j.cub.2003.10.031 14614828

[68] Shaw, R.J., Kosmatka, M., Bardeesy, N., Hurley, R.L., Witters, L.A., DePinho, R.A. et al. (2004) The tumor suppressor LKB1 kinase directly activates AMP-activated kinase and regulates apoptosis in response to energy stress. Proc. Natl Acad. Sci. U.S.A. 101, 3329–3335 10.1073/pnas.0308061100 14985505 PMC373461

[69] Woods, A., Dickerson, K., Heath, R., Hong, S.P., Momcilovic, M., Johnstone, S.R. et al. (2005) Ca^2+^/calmodulin-dependent protein kinase kinase-beta acts upstream of AMP-activated protein kinase in mammalian cells. Cell Metab. 2, 21–33 10.1016/j.cmet.2005.06.005 16054096

[70] Hawley, S.A., Pan, D.A., Mustard, K.J., Ross, L., Bain, J., Edelman, A.M. et al. (2005) Calmodulin-dependent protein kinase kinase-beta is an alternative upstream kinase for AMP-activated protein kinase. Cell Metab. 2, 9–19 10.1016/j.cmet.2005.05.009 16054095

[71] Hurley, R.L., Anderson, K.A., Franzone, J.M., Kemp, B.E., Means, A.R. and Witters, L.A. (2005) The Ca^2+^/calmoldulin-dependent protein kinase kinases are AMP-activated protein kinase kinases. J. Biol. Chem. 280, 29060–29066 10.1074/jbc.M503824200 15980064

[72] Momcilovic, M., Hong, S.P. and Carlson, M. (2006) Mammalian TAK1 activates Snf1 protein kinase in yeast and phosphorylates AMP-activated protein kinase in vitro. J. Biol. Chem. 281, 25336–25343 10.1074/jbc.M604399200 16835226

[73] Herrero-Martin, G., Hoyer-Hansen, M., Garcia-Garcia, C., Fumarola, C., Farkas, T., Lopez-Rivas, A. et al. (2009) TAK1 activates AMPK-dependent cytoprotective autophagy in TRAIL-treated epithelial cells. EMBO J. 28, 677–685 10.1038/emboj.2009.8 19197243 PMC2666037

[74] Jia, J., Bissa, B., Brecht, L., Allers, L., Choi, S.W., Gu, Y. et al. (2020) AMPK, a regulator of metabolism and autophagy, is activated by lysosomal damage via a novel galectin-directed ubiquitin signal transduction system. Mol. Cell 77, 951–969 10.1016/j.molcel.2019.12.028 31995728 PMC7785494

[75] Jenne, D.E., Reimann, H., Nezu, J., Friedel, W., Loff, S., Jeschke, R. et al. (1998) Peutz-Jeghers syndrome is caused by mutations in a novel serine threonine kinase. Nat. Genet. 18, 38–43 10.1038/ng0198-38 9425897

[76] Hemminki, A., Markie, D., Tomlinson, I., Avizienyte, E., Roth, S., Loukola, A. et al. (1998) A serine/threonine kinase gene defective in Peutz-Jeghers syndrome. Nature 391, 184–187 10.1038/34432 9428765

[77] Sanchez-Cespedes, M., Parrella, P., Esteller, M., Nomoto, S., Trink, B., Engles, J.M. et al. (2002) Inactivation of LKB1/STK11 is a common event in adenocarcinomas of the lung. Cancer Res. 62, 3659–3662 10.1158/2159-8290.CD-20-1353 12097271

[78] Ji, H., Ramsey, M.R., Hayes, D.N., Fan, C., McNamara, K., Kozlowski, P. et al. (2007) LKB1 modulates lung cancer differentiation and metastasis. Nature 448, 807–810 10.1038/nature06030 17676035

[79] Vara-Ciruelos, D., Russell, F.M. and Hardie, D.G. (2019) The strange case of AMPK and cancer: Dr Jekyll or Mr Hyde? Open Biol. 9, 190099 10.1098/rsob.190099 31288625 PMC6685927

[80] Lizcano, J.M., Göransson, O., Toth, R., Deak, M., Morrice, N.A., Boudeau, J. et al. (2004) LKB1 is a master kinase that activates 13 protein kinases of the AMPK subfamily, including the MARK/PAR-1 kinases. EMBO J. 23, 833–843 10.1038/sj.emboj.7600110 14976552 PMC381014

[81] Fogarty, S., Ross, F.A., Vara Ciruelos, D., Gray, A., Gowans, G.J. and Hardie, D.G. (2016) AMPK causes cell cycle arrest in LKB1-deficient cells via activation of CAMKK2. Mol. Cancer Res. 14, 683–695 10.1158/1541-7786.MCR-15-0479 27141100 PMC5390849

[82] Eichner, L.J., Brun, S.N., Herzig, S., Young, N.P., Curtis, S.D., Shackelford, D.B. et al. (2019) Genetic analysis reveals AMPK is required to support tumor growth in murine Kras-dependent lung cancer models. Cell Metab. 29, 285–302 10.1016/j.cmet.2018.10.005 30415923 PMC6365213

[83] La Montagna, M., Shi, L., Magee, P., Sahoo, S., Fassan, M. and Garofalo, M. (2021) AMPKalpha loss promotes KRAS-mediated lung tumorigenesis. Cell Death Differ. 28, 2673–2689 10.1038/s41418-021-00777-0 34040167 PMC8408205

[84] Rolf, J., Zarrouk, M., Finlay, D.K., Foretz, M., Viollet, B. and Cantrell, D.A. (2013) AMPKalpha1: a glucose sensor that controls CD8 T-cell memory. Eur. J. Immunol. 43, 889–896 10.1002/eji.201243008 23310952 PMC3734624

[85] Vara-Ciruelos, D., Dandapani, M., Russell, F.M., Grzes, K.M., Atrih, A., Foretz, M. et al. (2019) Phenformin, but not metformin, delays development of T cell acute lymphoblastic leukemia/lymphoma via cell-autonomous AMPK activation. Cell Rep. 27, 690–698 10.1016/j.celrep.2019.03.067 30995468 PMC6484776

[86] Zhou, G., Myers, R., Li, Y., Chen, Y., Shen, X., Fenyk-Melody, J. et al. (2001) Role of AMP-activated protein kinase in mechanism of metformin action. J. Clin. Invest. 108, 1167–1174 10.1172/JCI13505 11602624 PMC209533

[87] Evans, J.M., Donnelly, L.A., Emslie-Smith, A.M., Alessi, D.R. and Morris, A.D. (2005) Metformin and reduced risk of cancer in diabetic patients. BMJ 330, 1304–1305 10.1136/bmj.38415.708634.F7 15849206 PMC558205

[88] Kishton, R.J., Barnes, C.E., Nichols, A.G., Cohen, S., Gerriets, V.A., Siska, P.J. et al. (2016) AMPK is essential to balance glycolysis and mitochondrial metabolism to control T-ALL cell stress and survival. Cell Metab. 23, 649–662 10.1016/j.cmet.2016.03.008 27076078 PMC4832577

[89] Cerami, E., Gao, J., Dogrusoz, U., Gross, B.E., Sumer, S.O., Aksoy, B.A. et al. (2012) The cBio cancer genomics portal: an open platform for exploring multidimensional cancer genomics data. Cancer Discov. 2, 401–404 10.1158/2159-8290.CD-12-0095 22588877 PMC3956037

[90] Gao, J., Aksoy, B.A., Dogrusoz, U., Dresdner, G., Gross, B., Sumer, S.O. et al. (2013) Integrative analysis of complex cancer genomics and clinical profiles using the cBioPortal. Sci. Signal. 6, l1 10.1126/scisignal.2004088 PMC4160307 23550210

[91] Monteverde, T., Muthalagu, N., Port, J. and Murphy, D.J. (2015) Evidence of cancer promoting roles for AMPK and related kinases. FEBS J. 282, 4658–4671 10.1111/febs.13534 26426570

[92] The Cancer Genome Atlas Research Network (2014) Comprehensive molecular profiling of lung adenocarcinoma. Nature 511, 543–550 10.1038/nature13385 25079552 PMC4231481

[93] Speidel, D. (2015) The role of DNA damage responses in p53 biology. Arch. Toxicol. 89, 501–517 10.1007/s00204-015-1459-z 25618545

[94] Fu, X., Wan, S., Lyu, Y.L., Liu, L.F. and Qi, H. (2008) Etoposide induces ATM-dependent mitochondrial biogenesis through AMPK activation. PLoS One 3, e2009 10.1371/journal.pone.0002009 18431490 PMC2329593

[95] Hande, K.R. (1998) Etoposide: four decades of development of a topoisomerase II inhibitor. Eur. J. Cancer 34, 1514–1521 10.1016/S0959-8049(98)00228-7 9893622

[96] Sanli, T., Rashid, A., Liu, C., Harding, S., Bristow, R.G., Cutz, J.C. et al. (2010) Ionizing radiation activates AMP-activated kinase (AMPK): a target for radiosensitization of human cancer cells. Int. J. Radiat. Oncol. Biol. Phys. 78, 221–229 10.1016/j.ijrobp.2010.03.005 20615625

[97] Li, S., Lavagnino, Z., Lemacon, D., Kong, L., Ustione, A., Ng, X. et al. (2019) Ca(2+)-stimulated AMPK-dependent phosphorylation of Exo1 protects stressed replication forks from aberrant resection. Mol. Cell 74, 1123–1137 10.1016/j.molcel.2019.04.003 31053472 PMC6588484

[98] Vara-Ciruelos, D., Dandapani, M., Gray, A., Egbani, E.O., Evans, A.M. and Hardie, D.G. (2018) Genotoxic damage activates the AMPK-alpha1 isoform in the nucleus via Ca2+/CaMKK2 signaling to enhance tumor cell survival. Mol. Cancer Res. 16, 345–357 10.1158/1541-7786.MCR-17-0323 29133590

[99] Jiang, Y., Dong, Y., Luo, Y., Jiang, S., Meng, F.L., Tan, M. et al. (2021) AMPK-mediated phosphorylation on 53BP1 promotes c-NHEJ. Cell Rep. 34, 108713 10.1016/j.celrep.2021.108713 33596428

[100] Chen, Z., Wang, C., Jain, A., Srivastava, M., Tang, M., Zhang, H. et al. (2020) AMPK interactome reveals new function in non-homologous end joining DNA repair. Mol. Cell. Proteom. 19, 467–477 10.1074/mcp.RA119.001794 PMC7050103 31900314

[101] Jiang, Y., Cong, X., Jiang, S., Dong, Y., Zhao, L., Zang, Y. et al. (2021) Phosphoproteomics reveals AMPK substrate network in response to DNA damage and histone acetylation. Genom. Proteom. Bioinform. 10.1016/j.gpb.2020.09.003 PMC9880816 33607295

[102] Cheratta, A.R., Thayyullathil, F., Hawley, S.A., Ross, F.A., Atrih, A., Lamont, D.J. et al. (2022) Caspase cleavage and nuclear retention of the energy sensor AMPK-alpha1 during apoptosis. Cell Rep. 39, 110761 10.1016/j.celrep.2022.110761 35508122 PMC9108549

[103] Hawley, S.A., Ross, F.A., Gowans, G.J., Tibarewal, P., Leslie, N.R. and Hardie, D.G. (2014) Phosphorylation by Akt within the ST loop of AMPK-α1 down-regulates its activation in tumour cells. Biochem. J. 459, 275–287 10.1042/BJ20131344 24467442 PMC4052680

[104] Kazgan, N., Williams, T., Forsberg, L.J. and Brenman, J.E. (2010) Identification of a nuclear export signal in the catalytic subunit of AMP-activated protein kinase. Mol. Biol. Cell 21, 3433–3442 10.1091/mbc.e10-04-0347 20685962 PMC2947478

